# Mononuclear but Not Polymorphonuclear Phagocyte Depletion Increases Circulation Times and Improves Mammary Tumor-Homing Efficiency of Donor Bone Marrow-Derived Monocytes

**DOI:** 10.3390/cancers11111752

**Published:** 2019-11-08

**Authors:** Francis Combes, Alexandros Marios Sofias, Séan Mc Cafferty, Hanne Huysmans, Joyca De Temmerman, Sjoerd Hak, Evelyne Meyer, Niek N. Sanders

**Affiliations:** 1 Laboratory of Gene Therapy, Department of Nutrition, Genetics and Ethology, Faculty of Veterinary Medicine, Ghent University, Heidestraat 19, 9820 Merelbeke, Belgium; francis.combes@ugent.be (F.C.); Sean.McCafferty@ugent.be (S.M.C.); Hanne.Huysmans@ugent.be (H.H.); Joyca.DeTemmerman@ugent.be (J.D.T.); 2 Cancer Research Institute Ghent (CRIG), 9000 Ghent, Belgium; 3 Department of Circulation and Medical Imaging, Faculty of Medicine and Health Sciences, Norwegian University of Science and Technology (NTNU), 7030 Trondheim, Norway; alexandrosofias@outlook.com (A.M.S.); Sjoerd.Hak@ntnu.no (S.H.); 4 Department of Pathology, Bacteriology and Poultry Diseases, Faculty of Veterinary Medicine, Ghent University, Salisburylaan 133, 9820 Merelbeke, Belgium; 5 Department of Pharmacology, Toxicology and Biochemistry, Faculty of Veterinary Medicine, Ghent University, Salisburylaan 133, 9820 Merelbeke, Belgium; Evelyne.Meyer@ugent.be

**Keywords:** BMDM, depletion, phagocytes, competition, clodronate, intravital microscopy, fluorescence

## Abstract

Tumor associated macrophages are an essential part of the tumor microenvironment. Consequently, bone marrow-derived monocytes (BMDMs) are continuously recruited to tumors and are therefore seen as ideal delivery vehicles with tumor-targeting properties. By using immune cell depleting agents and macroscopic in vivo fluorescence imaging, we demonstrated that removal of endogenous monocytes and macrophages (but not neutrophils) leads to an increased tumor accumulation of exogenously administered BMDMs. By means of intravital microscopy (IVM), we confirmed our macroscopic findings on a cellular level and visualized in real time the migration of the donor BMDMs in the tumors of living animals. Moreover, IVM also revealed that clodronate-mediated depletion drastically increases the circulation time of the exogenously administered BMDMs. In summary, these new insights illustrate that impairment of the mononuclear phagocyte system increases the circulation time and tumor accumulation of donor BMDMs.

## 1. Introduction

Recruitment of immune cells to the tumor microenvironment followed by successful recognition of the tumor cells is essential for terminating tumor progression [1]. However, this fundamental process can also contribute to the level of tumor malignancy [2,3]. This dual role of the immune system in the development of a tumor has been described by the immunoediting theory and consists of three consecutive phases: elimination, equilibrium and escape. During the elimination phase, most of the tumor cells are killed by the immune system. In the next phase, the division rate of the remaining tumor cells and the tumor killing rate of the immune system are in equilibrium. The final phase starts when the immune system is no longer able to control the tumor cell growth [4]. Interestingly, this last stage of tumor development is also dependent on the infiltration of immune cells to form an immune suppressive and hence, tumor permissive environment. We can exploit this immune cell dependency of tumors by first collecting and loading immune cells with anti-cancer agents and subsequently infusing them back into cancer patients. In this regard, monocytic cells are an interesting cell type because of their tumor-homing characteristics [5] and the fact that monocytic cells contribute to the pool of tumor associated macrophages (TAMs), which can constitute up to 50% of solid tumor masses [6]. These TAMs mainly differentiate from circulating Ly6C+ CCR2+ inflammatory monocytes which, in turn, originate from the bone marrow [7,8]. Despite the strong reliance of solid tumors on recruiting circulating monocytes, we could previously not find an increased accumulation of injected monocytes in mouse mammary tumors compared to other myeloid cell populations [9]. We hypothesized that a tumor-homing competition between endogenous immune cells and exogenously administered immune cells may have masked inherent differences in tumor-homing capacities between different cell populations. As established solid tumors attract massive amounts of immune cells, it is likely that the tumor vasculature is congested with multiple populations of immune cells in the process of rolling, adhering and diapedesis. Endogenous immune cells can then be considered as direct competitors of systemically administered cell-based therapies for trafficking to the tumor microenvironment. In this study, we explored how biodistribution patterns of injected primary bone marrow-derived monocytes (BMDMs) are affected by various regimens that deplete endogenous immune cells. We demonstrated that depletion of macrophages and monocytes by clodronate (CLO) liposomes increased the accumulation of exogenously administered BMDMs in the tumor, lungs and spleen of mice. An increased accumulation of BMDMs was also seen in the lungs, liver and spleen after specific depletion of Gr-1-positive cells (i.e., Ly6C+Ly6G- monocytes and Ly6C+Ly6G+ neutrophils) [10]. However, anti-Ly6G-mediated depletion of endogenous neutrophils prior to BMDM administration did not significantly increase the tumor-homing of BMDMs, nor did this have a significant effect on the accumulation in other tissues. Furthermore, using a four color intravital microscopy procedure, we confirmed our macroscopic observations and found that CLO liposome pretreatment leads to an increased circulation time and diapedesis of injected BMDMs in tumors of living mice.

## 2. Results

**Figure 1:**
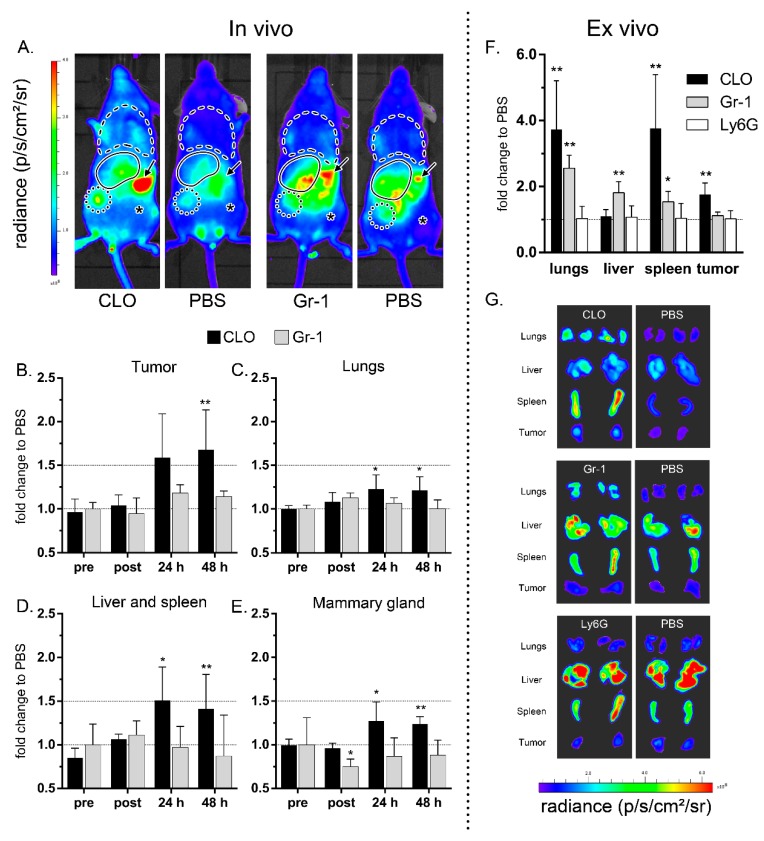
Depletion of the mononuclear phagocyte system increases the accumulation of exogenously injected bone marrow-derived monocytes (BMDMs) in the tumor and in other tissues. Mice were pre-treated with PBS, clodronate liposomes (CLO), anti-Gr-1 (Gr-1) or anti-Ly6G (Ly6G) antibodies 24 h before administration of 10^6^ DiD-labeled BMDMs. The total radiant efficiency was measured using macroscopic in vivo fluorescence imaging right before (pre) and directly after (post) BMDM injection as well as at 24 h and 48 h after injection. In (**A**), representative in vivo fluorescence images 48 h after BMDM injection are shown. The symbols represent the lungs (dashed line), the tumor (dotted circle), opposite mammary gland (asterisk), spleen (arrow) and presumed location of the liver (full line). (**B**–**E**) The fold change in BMDM accumulation immediately before (pre) and after (post) as well as at 24 and 48 h after injection of DiD-labelled BMDMs were calculated relative to control mice that were pretreated with PBS (y = 1). (**F**) Directly after the last in vivo measurement, the fluorescence of each individual organ was measured ex vivo and compared to the PBS pretreated control mice (y = 1). (**G**) Representative ex vivo fluorescence images of the tumors and dissected organs. The depletion experiments were independently conducted. This explains the variation in fluorescence between the PBS groups in G and in Figure S1D-F. The error bars represent the standard deviations (*n* = 6). * *p* < 0.05, ** *p* < 0.01 compared to the respective PBS control at each separate time point. Individual measurements can be found in Supplementary Figure S2.

Ex vivo engineered immune cells have been considered for anti-cancer immunotherapy and as cellular vehicles for targeted delivery of cargoes to tumors or other inflamed tissues [10,11,12,13,14]. However, the effect of endogenous immune cells on the tumor-homing and diapedesis of exogenous immune cells is not known. In this study, we pretreated (24 h beforehand) tumor-bearing mice with clodronate (CLO) liposomes, anti-Gr-1 or anti-Ly6G antibodies to assess the role of the mononuclear phagocytic system and specific subsets of myeloid cells on the distribution of exogenously administered DiD-labeled BMDMs in living mice. Controls regarding the validity of the conducted experiments include initial tumor size, antibody specificity, immune cell depletion and BMDM labeling efficiency (Supplementary Figure S1). CLO liposomes-mediated depletion increased the tumor accumulation of BMDMs 24 h (*p* = 0.0931) and 48 h (*p* = 0.0087) after their intravenous injection (Figure 1A,B,F,G and Supplementary Figure S2J). In contrast, pretreatment with anti-Gr-1 antibodies did not significantly increase the accumulation of BMDMs in the tumors (Figure 1A,B,F,G and Supplementary Figure S2K). Depletion with CLO liposomes also slightly increased the amount of DiD-labeled BMDMs in the opposite tumor-free mammary gland (*p* = 0.026 at 24 h and *p* = 0.0022 at 48 h), while anti-Gr-1 depletion had no effect or even decreased the fluorescence in this region (*p* = 0.0173 directly after injection in anti-Gr-1-pretreated mice) (Figure 1E and Supplementary Figure S2G,H).

We also investigated the BMDM accumulation patterns in the lungs and the liver-and-spleen region. Immediately after their intravenous injection, the BMDMs mainly accumulated in the lungs of immune cell depleted and non-depleted mice (Supplementary Figure S2A–C). After 24 h, a redistribution of the BMDMs from the lungs to the tumor and liver-and-spleen region was apparent in all the mice (Supplementary Figure S2A–F). Nevertheless, significantly more BMDMs remained in the lungs of mice pretreated with CLO liposomes compared to mice pretreated with PBS (*p* = 0.00411 at 24 h and at 48 h) (Figure 1C). Moreover, ex vivo imaging of the lungs after euthanasia showed that both CLO liposomes and anti-Gr-1-pretreated mice still contained significantly more BMDMs than PBS control mice (*p* = 0.0022 and *p* = 0.0043, respectively; Figure 1F). As mentioned, BMDMs started to accumulate in the liver-and-spleen region of all mice after 24 h and even more BMDMs accumulated in this region when mice were pretreated with CLO liposomes (*p* = 0.0026 at 24 h and *p* = 0.0043 at 48 h, Figure 1D and Supplementary Figure S2). Interestingly, ex vivo imaging of both organs separately demonstrated that only the spleen of CLO liposomes-pretreated mice contained increased BMDMs compared to the PBS controls (*p* = 0.0022) whereas anti-Gr-1-treated mice exhibited increased BMDMs in both the liver (*p* = 0.0043) and the spleen (*p* = 0.0173, Figure 1F).

In a subsequent experiment, we evaluated whether depletion of endogenous cells that are not related to the injected cells could influence the BMDM biodistribution. To that extent, we specifically depleted neutrophils in tumor-bearing mice using anti-Ly6G antibodies (Supplementary Figure S1J). Interestingly, neutrophil depletion did not significantly alter the in vivo tumor-homing of BMDMs, nor their accumulation in other tissues (Supplementary Figure S2). Likewise, ex vivo imaging of excised tumors and organs of anti-Ly6G pretreated mice demonstrated that the number of fluorescent BMDMs in these tissues was not different from the PBS-pretreated control mice (Figure 1F, Supplementary Figure S3).

**Figure 2:**
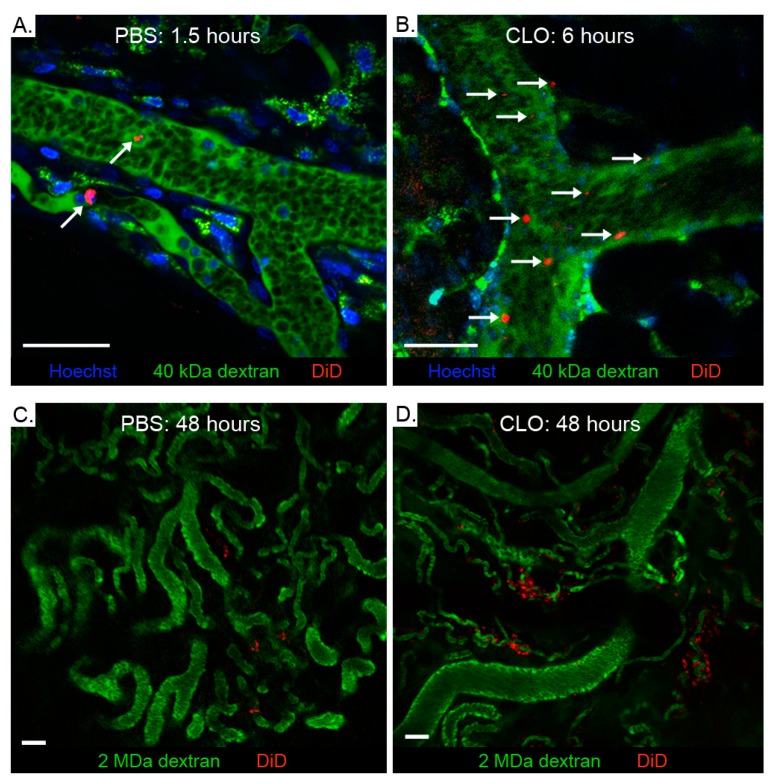
Depletion of the mononuclear phagocyte system prolongs the circulation of exogenously administered BMDMs. Mice were first pretreated with either PBS or CLO liposomes. After 24 h, 10^6^ DiD-labeled BMDMs were injected and their appearance in the tumor microenvironment was monitored by intravital microscopy. In PBS-pretreated mice (**A**), only a few circulating DiD-labeled BMDMs (red, arrows) can be seen 90 min after their administration, whereas 6 h after their administration many BMDMs were still detected in the blood of CLO liposomes-pretreated mice (**B**) (see also accompanied Supplementary video S1). Hoechst (blue) and 40 kDa TMR-dextran (green) were systemically injected before imaging to discriminate endogenous macrophages (green) from exogenous BMDMs (red). Residual 40 kDa TMR-dextran in the blood within the first hours after injection allows visualization of the blood vessels as well. Two days after their injection, considerably more BMDMs can be seen in the tumors of mice pretreated with CLO liposomes (**D**) compared to PBS pretreated control mice (**C**). Most of these extravasated BMDMs are present near capillaries. The green color in (**C**) and (**D**) originates from 2 MDa FITC-dextran, a fluorescent vascular tracer. Scale bars: 50 µm.

**Figure 3:**
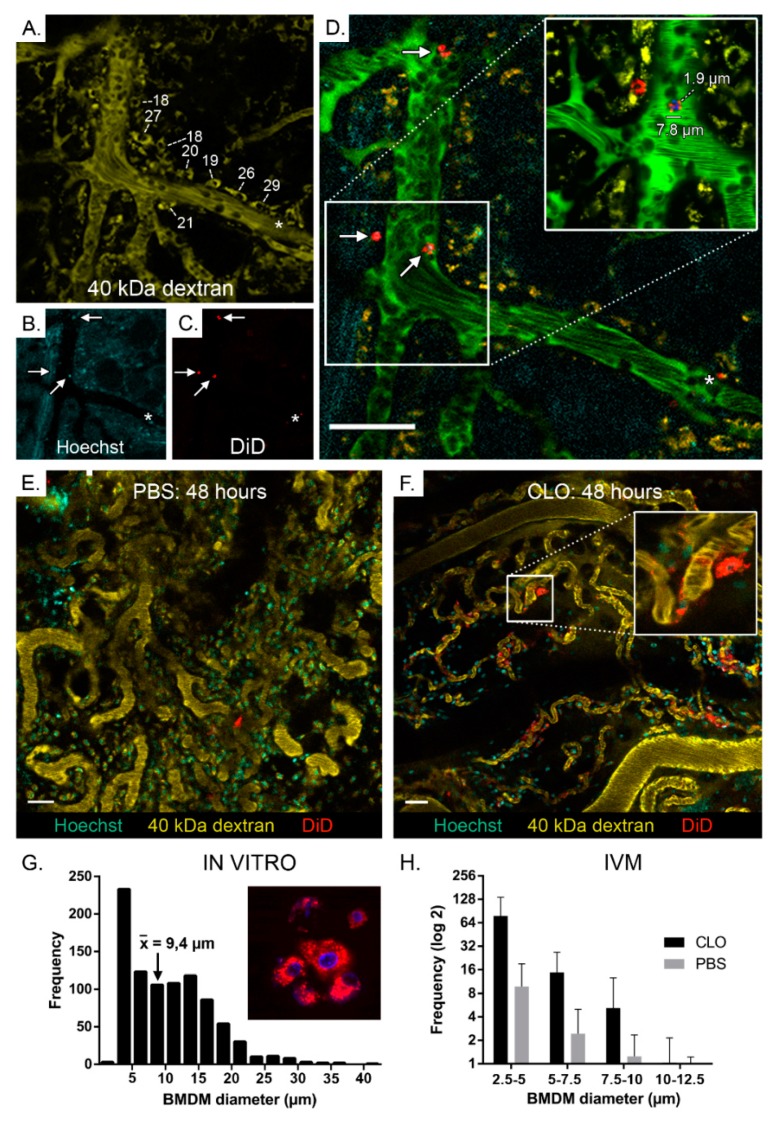
Depletion of the mononuclear phagocyte system increased the accumulation of BMDMs in the tumor interstitium. Mice were first pretreated with either PBS or CLO liposomes. After 24 h, 10^6^ BMDMs that were labeled with both DiD (red) and Hoechst (blue) were injected in mice pretreated with CLO liposomes. After 60–90 min, the appearance of these double labeled BMDMs (white arrows in (**A**–**D**) in the tumor microenvironment was imaged by intravital microscopy. The insert in panel D demonstrates that non-uniformly labeled BMDMs can appear as small fluorescent speckles. The used labeling strategy allows separation of injected Hoechst/DiD-positive BMDMs (7.8 µm) from larger (18–29 µm) 40 kDa TMR-dextran-positive endogenous phagocytes (yellow). The white numbers in panel A represent the long diameter (in µm) of several endogenous macrophages lining the blood vessels. The asterisk in panels A–D shows an endogenous phagocyte suspected of engulfing DiD-labeled cell fragments (See also Supplementary video S3). Panel D was obtained by merging panels (**A**–**C**) with an additional layer of 2 MDa FITC-dextran (green) to delineate the blood vessels. (**G**) Size distribution of cultured DiD-labeled BMDMs. BMDMs were labeled with DiD and after 2 days of culture, confocal fluorescence images were taken and the size distribution of the cells was analyzed using Image J (bin size of 2.5 µm). A mean BMDM diameter of 9.4 µm was determined and fluorescent particles smaller than 2.5 µm were identified as background noise. (**H**) The size distribution of exogenously administered BMDMs that arrived in the tumor was quantified by automated particle counting on IVM images such as panels (**E**) and (**F**). The data clearly show an increased accumulation of BMDMs in tumors of mice that were pretreated with CLO liposomes. Scale bars: 50 µm; the error bars represent standard deviation, *n* = 3.

Macroscopic in vivo fluorescence imaging is often used to monitor the in vivo migration of cells. However, this technique does not give information about the diapedesis efficiency as it cannot reveal the microscopic location of exogenous cells in tissues. Therefore, intravital microscopy (IVM) was used to further evaluate the increased tumor accumulation of BMDMs after pretreatment with CLO liposomes (Supplementary Figure S4). We developed an IVM protocol that enabled simultaneous visualization of nucleated cells, blood vessels, endogenous macrophages and the intravenously injected DiD-labeled BMDMs. This protocol allowed us to discriminate intact BMDMs from endogenous macrophages that internalized BMDM fragments. Circulating immune cells in the blood vessels of tumors were visualized by employing fast imaging (0.6 s/frame), allowing us to identify differences regarding BMDM clearance between PBS- and CLO liposomes-pretreated mice. Exogenous BMDMs were rapidly (<30 min) cleared in PBS-pretreated mice (Figure 2A and Supplementary video S1) and only a few BMDMs remained in circulation after 1.5 h. In contrast, in mice pretreated with CLO liposomes, exogenous BMDMs displayed a prolonged blood circulation with many cells visible 6 h as well as 48 h after BMDM injection (Figure 2B and Supplementary videos S1 and S2). Importantly, pre-treatment with CLO liposomes also increased the diapedesis (extravasation) of the BMDMs at the tumor. This observation was made as soon as a few hours post injection and was even more evident at 48 h post injection, where much more fluorescent BMDMs were detected in the extravascular compartment of CLO liposomes-pretreated mice (Figure 2C,D). These extravasated BMDMs were mainly found around small blood vessels. In addition to extravasated DiD-labeled BMDMs, we also found BMDMs that stuck to the blood vessel walls and were presumably in the process of diapedesis (Supplementary videos S2, S3 and S4). To further confirm that the DiD-labeled structures were intact BMDMs, double labeled BMDMs (DiD and Hoechst) were injected together with an endogenous macrophage tracer (40 kDa TMR-dextran) in tumor-bearing mice (Figure 3A–F). Clearly intact BMDMs (red and blue colored cytosol and nucleus, respectively) (Figure 3F insert) as well as intact BMDMs with a more speckled fluorescence pattern (Figure 3D insert) could be observed by IVM (see also Supplementary video S3). This experiment also demonstrated that endogenous macrophages are frequently positioned in close proximity to blood vessels and that these cells have a more spread-out morphology (longest diameter ranging from 18 to 29 µm) compared to the injected BMDMs (about 8–10 µm; Figure 3A–D). Moreover, in addition to DiD+/Hoechst+ BMDMs, some circulating cells appear to be only Hoechst+ or only DiD+. This can be attributed to imperfect staining or imaging artifacts. Smaller DiD+ particles were also seen in association with endogenous macrophages (asterisk in Figure 3A–D and Supplementary video S3), indicating horizontal dye transfer. Image analysis was performed on the IVM data (Figure 3H) to quantify the differences in tumor accumulation of BMDMs in PBS- and CLO liposomes-pretreated mice. This revealed a clear trend in increased accumulation of exogenously administered BMDMs in tumors of CLO liposomes-pretreated mice compared to tumors of PBS-pretreated mice. The increased fluorescence originated from uniformly as well as irregularly labeled BMDMs (mean in vitro diameter of 9.4 µm; (Figure 3F,H).

## 3. Discussion

The growing interest in cell-based tumor therapies has revealed critical knowledge gaps in our understanding of the basic principles of immune cell recruitment to the tumor microenvironment [15,16,17]. Our investigation aimed to address one of these gaps by evaluating how the presence of endogenous immune cells affects the recruitment of exogenously administered immune cells to tumors. In more detail, we studied how depletion of specific myeloid leukocyte populations influenced the tumor-homing and tissue distribution of exogenous bone marrow-derived monocytes (BMDMs). By combining advanced macroscopic and microscopic in vivo fluorescence imaging techniques, we revealed that depletion of macrophages and monocytes increased the blood circulation time, tumor-homing and extravasation of BMDMs at the tumor. In contrast, depletion of neutrophils, which do not belong to the monocytic lineage, did not increase the tumor accumulation of BMDMs. This suggests that the tumor accumulation of exogenous immune cells might be impeded by endogenous cells of the same lineage.

Macroscopic in vivo fluorescence imaging is limited by the tissue penetrance of the used excitation light and should therefore be complemented with ex vivo measurements of excised organs. In addition, these macroscopic techniques do not allow discrimination of intact cells from labeled cell fragments and do not provide information about the exact histological location of the emitted fluorescence. Therefore, we complementary used intravital microscopy (IVM) to confirm our findings and to further investigate the effect of CLO liposomes on the migration process at a microscopic level. Although many of the extravascular DiD-positive cells appeared uniformly stained and thus intact, smaller-sized fluorescent speckles could also be seen. Through injection of Hoechst+/DiD+ double labeled BMDMs, we demonstrated that these speckles can originate from non-uniformly stained BMDMs (Figure 3D). However, endogenous macrophages that phagocytosed DiD-labeled cell fragments (e.g., apoptotic bodies) can also appear as fluorescent speckles. Injecting 40 kDa TMR-dextran before imaging allowed us to distinguish injected DiD-positive BMDMs from secondary labeled DiD+/TMR+ phagocytes on IVM images and movies. Moreover, using image analysis software, we were able to quantify the BMDMs in the tumors and to determine their size distribution (Figure 3H). The data obtained from IVM confirmed that pretreatment with CLO liposomes increased the extravascular accumulation of BMDMs in the tumor. Moreover, IVM also revealed a drastically prolonged BMDM circulation time in mice pretreated with CLO liposomes (Figure 2A,B). This can explain the increased number of DiD-labeled BMDMs seen at the tumor-free mammary gland of mice pretreated with CLO liposomes (Figure 1E). Weisser et al. (2012) made a similar observation in an experimental colitis model [18]. In their model, gut macrophages were depleted via intraperitoneal injection of CLO liposomes and polarized BMDMs were intravenously injected. Subsequent histological staining demonstrated that the exogenously administered BMDMs repopulated the inflamed colon, indicating that the increased infiltration of BMDMs after a depletion regimen can be applied to different inflammatory-based settings. However, these imaging techniques only confirm the presence of injected cells in the tumor. More elaborate research is needed to adequately assess their functional properties after arrival in the tumor. While the possibility of endogenous immune cell competition on the recruitment efficiency of adoptively transferred myeloid cells has been briefly mentioned before [19], to our knowledge, the effect of endogenous myeloid cell depletion on the migration of exogenous BMDMs towards the tumor has never been studied.

The exogenous BMDMs did not only accumulate in the tumors. In vivo macroscopic fluorescence imaging revealed that a large portion of the intravenously injected BMDMs immediately accumulate in the lungs (Supplementary Figure S2). Given that the lungs receive the complete cardiac output and exhibit narrow capillary diameters, it is not surprising that they capture a substantial part of the injected cells [20]. Yet, we did not observe any symptoms of respiratory distress in this study, nor did we see such symptoms when mice were imaged over a period of 20 days [9]. Over the following days, a redistribution of the BMDMs from the lungs to the tumor and liver-and-spleen region occurred. The latter organs are typical accumulation sites due to their large size and vascular volume [21]. Interestingly, this reallocation to other organs occurred significantly more slowly in CLO liposomes-depleted mice than in PBS or antibody (anti-Gr-1 or anti-Ly6G) depleted mice. This can be explained by either a stronger and longer adherence of injected BMDMs in the lungs or by the prolonged circulation of BMDMs in CLO liposomes-pretreated mice (Figure 2B). Because the lungs act as a margination pool for leukocytes [22,23], it is not a surprise that the BMDMs are captured by this organ.

In vivo, anti-Gr-1 pretreatment demonstrated no differences compared to PBS mice. However, ex vivo imaging of the individual organs revealed significantly higher BMDMs in the lungs, liver and spleen, but not the tumors of anti-Gr-1 pretreated mice (Figure 1B–F). Still, we have to interpret these data with caution. Antibodies are known to have a longer serum half-life (around 1–3 weeks) than PEGylated CLO liposomes (about 20 min). Therefore, these antibodies were most likely still present when the donor BMDMs were injected. Binding of the anti-Gr-1 antibodies to the BMDMs would then lead to opsonisation and enhanced clearance of the injected Ly6C-positive BMDMs in mice pretreated with antibodies but not in CLO liposomes-pretreated mice (Figure 1F) [9,24,25,26]. Our former experiments have indicated that a large percentage of the BMDMs obtained with our protocol are Ly6C-positive [9], making this theory plausible. Furthermore, the significantly lower fluorescence signal of the BMDMs in the tumor-free mammary gland of anti-Gr-1 pretreated mice is indicative of a higher clearance of BMDMs and hence underpins the hypothesis that residual anti-Gr-1 antibodies also depleted the exogenous BMDMs (Figure 1E). Of note, this critical remark does not apply to the anti-Ly6G-mediated depletion since BMDMs do not express this marker [10].

Removal of endogenous macrophages by CLO liposomes increased the circulation time of exogenous BMDMs (Figure 2B). Our experiments did not allow the determination of whether the increased presence of tumor-associated BMDMs after pretreatment with CLO liposomes resulted from a decline in endogenous competition for vascular binding sites or whether it was merely due to more BMDMs circulating. The feasibility to deplete immune cells in humans has been demonstrated in the field of, e.g., CAR T cell therapy, where the current view is that endogenous T cells might act as “cytokine sinks”, i.e., they deprive the injected CAR T cells from the cytokines that are necessary for adequate activation and proliferation [27,28,29]. Therefore, a common approach to increase the clinical efficacy of CAR T cell therapy is to perform lymphodepletion prior to infusing the genetically modified lymphocytes. In analogy with the lymphodepletion regimens before CAR T cell therapy, one can wonder whether it is sensible to administer BMDMs before or after monocyte-depleting nonmyeloablative anti-cancer treatments [30,31,32].

Macrophages play an important role in efferocytosis, the removal of dead or dying blood cells. Therefore, it is tempting to speculate that a portion of the exogenous BMDMs are phagocytosed by endogenous macrophages and that depletion of these phagocytes prevented compromised exogenous BDMDs from being cleared from the circulation. Compromised and senescent cells are cleared by phagocytic cells after recognition of “eat me” cell surface markers (e.g., phosphatidylserine [33,34] or oxidized lipids [35]) on apoptotic cells. We hypothesize that the ex vivo manipulation of the BMDMs induced the expression of clearance markers in a fraction of the cells, despite demonstrating above 95% cell viability upon injection (trypan blue). Ideally, the efficiency of future adoptive cell transfer experiments can be increased by first clearing this ill-fated population before administration. Alternatively, masking phosphatidylserine [36] or incorporation of “don’t eat me” signals such as CD47 can be used for therapeutic purposes [37].

In the broad context of drug delivery, immune cells are interesting drug delivery vehicles as they are actively recruited to sites of inflammation (e.g., the tumor microenvironment). They can be loaded with therapeutic agents or transfected with DNA or mRNA vectors that encode a therapeutic protein [11,19]. Furthermore, the release of the therapeutic cargo can initiate tumor cell destruction, which leads to more inflammation and further recruitment of the cell vehicles to the site of interest [11]. A second argument of using cells as delivery vehicles over, e.g., nanoparticles, is that the cells can actively penetrate the tumor tissue whereas nanoparticles are restricted to passive diffusion and hence, are often impeded by the high interstitial pressure seen in many solid tumors [38,39]. However, a disadvantage of cell-based drug delivery systems is their complicated production process and the need for cells that are not recognized as foreign by the patient. From our data, it appears that cell recognition might also apply to autologous cells that haven undergone manipulation. Considering the increased interest in immune cell therapies over the last decade, addressing this lack in understanding could potentially help improve the unresolved recruitment problem which many of these therapies are facing.

## 4. Materials and Methods

### 4.1. Animals

All the procedures in this study were approved by the Ethical Committee of the Faculty of Veterinary Medicine and the Faculty of Bioscience Engineering of Ghent University, Belgium (EC 2015/100) and the Norwegian Food Safety Authority (19/26187). Female Balb/cJRj mice, aged 6–8 weeks, were purchased from Janvier Labs (Paris, France) and housed in a temperature and humidity-controlled room while being kept on a 12 h:12 h reverse light/dark cycle. Ad libitum access to low-fluorescence food (Envigo, Boxmeer, Netherlands, #T.2018.12) and water was provided. Mice were ear marked and randomly assigned to experimental conditions. All manipulations were performed on a heated platform and under general anesthesia using 5% isoflurane (Zoetis, Louvain-la-Neuve, Belgium, #B506) at 4 L/min oxygen for induction and 1.5–2% isoflurane at 0.5–1 L/min oxygen for maintenance.

### 4.2. Tumor Model

Luciferase-positive 4T1 mammary carcinoma cells were cultured in complete medium consisting of DMEM/F12 (Thermo Fisher, Aalst, Belgium, #21041-025) supplemented with 10% heat inactivated FBS (VWR, Leuven, Belgium, #S181H-500) and 1% penicillin/streptomycin (Thermo Fisher, Aalst, Belgium, #15070-063). After at least 3 passages, cells were trypsinized and washed twice in Dulbecco’s Phosphate-Buffered Saline (Thermo Fisher, Aalst, Belgium, #14190-144). Subsequently, 1 × 10^5^ cells in 100 µL DPBS, were injected in the 4th right fat pad using a 29 G insulin syringe (Terumo, Leuven, Belgium, #BS05M2913). Tumor growth was verified by administering 150 µL D-luciferin (15 mg/mL DPBS) (Goldbio, St-Louis (MO), USA, #LUCK-1G) subcutaneously followed by bioluminescence imaging after 10 min with an IVIS Lumina II system (PerkinElmer). Cell migration experiments were initiated 10 days post tumor inoculation. At this time point, tumors reached an average diameter of 4.0–4.8 mm. This average tumor diameter was obtained by measuring both perpendicular diameters twice and then averaging the total of 4 measurements.

### 4.3. Bone Marrow-Derived Monocytes

Bone marrow cells were isolated from female Balb/cJRj mice according to the method described by Amend et al. (2016) [40]. Mice were induced with isoflurane and sacrificed via cervical dislocation. Next, femurs and tibias were dissected, sterilized 10 s in 70% Disinfectol (Chem-lab NV, Zedelgem, Belgium, #CL00.0112.2500) and rinsed in sterile DPBS before snapping the bones in half and transferring these to punctured 0.5 mL Eppendorf tubes that were placed in empty 1.5 mL Eppendorf tubes. After centrifugation for 15 s at 10,000× *g*, recovered pellets were resuspended 40 s in 150 µL ACK RBC lysis buffer (Thermo Fisher, Aalst, Belgium, #A10492-01) and neutralized by adding 1 mL complete medium. Finally, these cells were centrifuged for 5 min at 400× *g* and resuspended in complete medium (DMEM/F-12, 10% FBS, 1% penicillin/streptomycin).

Bone marrow-derived monocytes (BMDMs) were obtained by seeding 5 ml RBC-depleted bone marrow cells (1–2 × 10^6^ cells/mL) in complete medium supplemented with 20 ng/mL M-CSF (VWR, Leuven, Belgium, #21-8983-U010). Cells were cultured for 5 days in an untreated 9 cm petri dish (VWR, Leuven, Belgium, #391-0457) to prevent adherence-induced differentiation [41]. Fresh culture medium was added 3 days post-seeding (3 mL per well). After 5 days, cells in suspension were collected and adherent cells were detached and collected by 30 min detachment in the protease/collagenase cocktail Accutase^TM^ (Biolegend, Koblenz, Germany, #424301) and subsequent neutralization by addition of warm complete medium. Adherent cells and cells in suspension were pooled.

### 4.4. Cell Labeling

Labeling with Vybrant DiD (Thermo Fisher, Aalst, Belgium, #V22887) was performed by adding 1 µL of 2 mM stock dye to cells suspended in 200 µL DPBS at a concentration of 5 × 10^6^/mL. After vortexing, the cells were incubated for 20 min at 37 °C in darkened 15 mL tubes. Subsequently, they were centrifuged for 5 min at 400× *g* and resuspended in warm complete medium. Next, cells were washed twice in DPBS and resuspended in appropriate downstream buffer.

### 4.5. Systemic Injection

One hour before administration, clodronate (CLO) liposomes or control liposomes (Liposoma, Amsterdam, The Netherlands, #CP-005-005) were kept at room temperature. Per mouse, 200 µL liposomes (50 µg/g) was administered retro-orbitally after vortexing, using a 29 G insulin syringe (VWR, Leuven, Belgium,). Likewise, labeled BMDMs were vortexed prior to injection with a 29 G insulin syringe. Unless noted otherwise, 100 µL of cells suspended in DPBS were injected in the right orbital plexus of anesthetized mice at a concentration of 10 × 10^6^/mL [42,43]. Anti-Gr-1 (Bioxcell, Lebanon, USA, #BE0075) or anti-Ly6G (Bioxcell, Lebanon, USA, #BE0075-1) depleting monoclonal antibodies were administered intraperitoneally at a dose of 250 µg per mouse in 100 µL DPBS [44].

### 4.6. Flow Cytometry

Cells were suspended at 1 × 10^6^/mL in 200 µL staining buffer (DPBS + 2%FBS + 2mM EDTA) followed by adding 0.5 µL anti-CD16/32 FcR blocking antibodies (BD, #553142) [45]. After 10 min incubation at 4 °C additional fluorescently labelled antibodies against the selected markers were added and the cells were further incubated at 4 °C for 15 min. Subsequently, they were washed by adding 1 mL staining buffer per dark 1.5 mL Eppendorf tube and centrifuged for 5 min at 400× *g*. The resulting pellet was resuspended in 200 µL staining buffer. The DNA intercalating dye 7-AAD (Biolegend, Koblenz, Germany, #420403) was used to exclude dead cells. A weekly calibrated and validated C6 Accuri (BD) or Cytoflex (Beckman Coulter, Brea, CA, USA) flow cytometer was used for acquisition. BD Accuri C6 software (version 1.0.264.21, San Jose, CA, USA) was used for analysis. The following selected antibodies were used (from Biolegend, San Diego, CA, USA): anti-Ly6C-FITC (#128005), anti-CD11b (#101211). Anti-F4/80-R-PE was ordered from Biorad (#MCA497PET). Doublets, debris and dead cells were excluded in all analyses.

### 4.7. In Vivo and Ex Vivo Fluorescence Imaging

In vivo fluorescence imaging of the DID-labeled cells was performed with an IVIS Lumina II system (PerkinElmer, Waltham, MA, USA) using the 740/780 nm filter pair. All the mice were ventrally shaven from the cervical to the pubis region before imaging. The accumulation of the labeled cells in the tumor, lungs, liver and spleen was quantified using the total radiant efficiency (p/s/µW/cm²). Reported values were normalized to the background fluorescence of each individual mouse before injection of BMDMs. Since spleen mobility can cause spleen and liver to overlap in vivo, the fluorescence of these organs was combined into one region of interest (ROI). ROI dimensions were kept constant for all the mice.

### 4.8. Intravital Microscopy

Female wild type Balb/c mice were orthotopically inoculated in the 4th mammary fat pad with 1–1.5 × 10^6^ 4T1 cells in 100 µL PBS or 1:1 0.9% NaCl:matrigel (Corning, Corning, NY, USA, #354262). Ten to fourteen days later, mice were anesthetized (subcutaneous injection with a mixture of fentanyl (0.05 mg/kg), medetomidine (0.5 mg/kg), midazolam (0.5 mg/kg) and water (2:1:2:5) at a dose of 0.1 mL per 10 gram of body weight) and their tumors were carefully detached as a “skin flap” from the abdominal wall without damaging the blood vessels. After the fat tissue surrounding the tumor was removed, the “skin flap” with the tumor was mounted on a custom-made imaging platform and then positioned on a heated (30 °C) microscope stage (Supplementary Figure S4). Twenty-four hours before imaging, mice were pretreated with either CLO liposomes or PBS. In addition to the DiD-labeled BMDM injection, the mice were also intravenously injected with 100 µL Hoechst 33342 (Sigma, #B2251-100MG, 2 mg/mL), 40 kDa TMR-dextran (#42874-1G, Sigma, 10 mg/mL) and/or 2 MDa FITC-dextran (#FD2000S, Sigma, 10 mg/mL). In the double labeling experiments, BMDMs were first labeled with Hoechst (100 ng/mL, 45 min at 37 °C) followed by labeling with DiD (20 min at 37 °C) and neutralizing by adding 2 volumes of complete medium. The stained BMDMs were washed twice in PBS before injection. Intravital microscopy was performed on a Leica SP8 confocal microscope (Leica microsystems, Wetzlar, Hesse, Germany) using a 20 × 0.75 air objective. The system is equipped with an external UV laser, a tunable white light laser and a tunable band pass detection system allowing the detection of four colors simultaneously: Hoechst (405/430–470 nm); GFP or FITC-dextran (488/505–520 nm); TMR-dextran (565/575–625 nm); DiD-BMDMs (644/654–700 nm). Data acquisition was performed with Leica LASX software. Image J (FIJI) version 1.52n was used for the image assembly and quantification of fluorescence occurred as follows: channel 3 (endogenous phagocytes labeled with TMR-dextran) was first subtracted from channel 4 (DiD-BMDMs) to exclude DiD-fluorescence associated with phagocytosis. Next, a black and white threshold was set, the image was converted to a binary mask and watershed was applied. Lastly, the number of particles and the areas above 1 µm^2^ were determined via the analyze particles command. Particle diameters were determined via the area of a circle (A = π·r^2^).

### 4.9. Statistics

Statistics were performed in Prism Graphpad (version 6.01, Graphpad Software, San Diego, CA, USA). Unless otherwise specified, two-way non-parametric Mann–Whitney rank tests were used. *p*-values lower than 5 percent were used to indicate statistical significance. The reported values represent averages ±standard deviation (SD).

## 5. Conclusions

Taken together, our results indicate that depletion of the mononuclear phagocyte system via CLO liposomes significantly increases the circulation time of systemically injected BMDMs and that a higher presence of these BMDMs can be seen in the tumor, the lungs and the spleen after 2 days. In contrast, depletion of endogenous immune cells that belong to another immune cell lineage than the exogenously administered BMDMs did not affect their tumor accumulation. We hypothesize that a subset of BMDMs expose “eat me” signals on their surface resulting from their ex vivo manipulation. By depleting the mononuclear phagocytic system, we might have prevented these tagged cells being massively removed upon injection.

## Supplementary Materials

The following are available online at https://www.mdpi.com/2072-6694/11/11/1752/s1, Figure S1: The initial tumor sizes and the injected doses of DiD-labeled BMDMs in each immune cell depletion group and respective control group were determined to ensure that changes in tumor homing of BMDMs were not biased by significant differences in tumor size or injected BMDM dose. In addition, blood was collected via cardiac puncture to verify functional depletion via flow cytometry. Figure S2: Total radiant efficiency (p/s/cm²/sr) measured by in vivo macroscopic fluorescence imaging at different organs and the tumor immediately before (pre) and after (post) DiD-BMDM injection as well as at 24 h and 48 h after injection of DiD-labeled BMDMs. Figure S3: Total radiant efficiency (p/s/cm²/sr) of tumors and organs excised from mice euthanized 48 h after injection of DiD-labeled BMDMs. Figure S4: Setup of the intravital microscopy (IVM) procedure. Video S1: Real time visualization of circulating DiD-labeled BMDMs in mammary fat pad tumors of PBS and CLO liposomes pretreated mice. Video S2: Real-time visualization of long-term circulating DiD-labeled BMDMs in mammary fat pad tumors of CLO liposomes-pretreated mice. Video S3: Z-stack (starting superficial) through a mammary fat pad tumor of a CLO liposomes-pretreated mouse that was injected with Hoechst (blue)/DiD (red) double labeled BMDMs. Video S4: Z-stacks (starting superficial) through mammary fat pad tumors of PBS- and CLO liposomes-pretreated mice that were injected with DiD-labeled BMDMs.
