# Supplementary material for: Mononuclear but Not Polymorphonuclear Phagocyte Depletion Increases Circulation Times and Improves Mammary Tumor-Homing Efficiency of Donor Bone Marrow-Derived Monocytes
Source: Cancers (Basel). 2019 Nov 8;11(11):1752. doi: 10.3390/cancers11111752 (PMC6896201; doi:10.3390/cancers11111752)
Supplement: Supplementary file 1 [file cancers-11-01752-s001.zip › cancers-616281-supplementary-figures.pdf]

cancers

MDPI

Article

Mononuclear but not Polymorphonuclear Phagocyte Depletion Increases Circulation Times and Improves Mammary Tumor-Homing Efficiency of Donor Bone Marrow-Derived Monocytes

Francis Combes [1,2], Alexandros Marios Sofias [3], Séan Mc Cafferty [1,2], Hanne Huysmans [1], Joyca De Temmerman [1,4], Sjoerd Hak [3], Evelyne Meyer [2,5] and Niek N. Sanders [1,2,*]

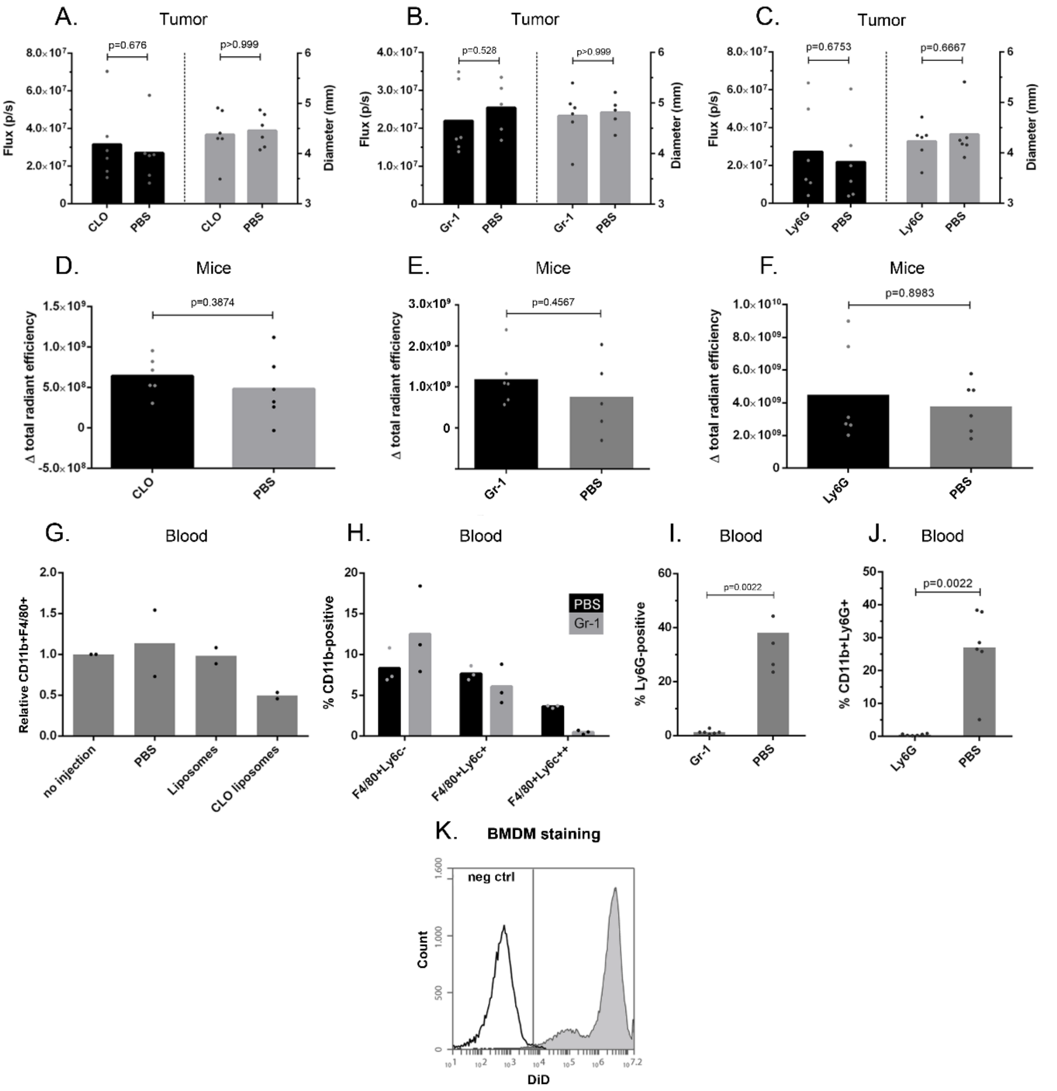

Figure S1. The initial tumor sizes and the injected doses of DiD-labeled BMDMs in each immune cell depletion group and respective control group were determined to ensure that changes in tumor homing of BMDMs were not biased by significant differences in tumor size or injected BMDM dose. In addition, blood was collected via cardiac puncture to verify functional depletion via flow cytometry. (**A–C**): Twenty-four hours before injection of respectively clodronate liposomes (CLO), anti-Gr-1 antibodies (Gr-1) and anti-Ly6G antibodies (Ly6G), tumor sizes (right Y-axis) and tumor bioluminescence (left Y-axis) were determined to be statistically equal to each respective PBS control group (n = 6). (**D–F**): After injection of 10^6 DiD-labeled BMDMs in each mouse, no significant

difference in the number of administered DiD-BMDMs could be detected ($n = 6$). (**G**): Relative number of CD11b+F4/80+ events in cardiac blood collected 24 hours after injection of 200 µL PBS, control liposomes or CLO liposomes. Flow cytometry demonstrated that CLO liposomes reduced the number of CD11b+F4/80+ monocytic cells with 60% ($n = 2$). (**H**): Percentage of CD11b+F4/80+Ly6C+/- events in cardiac blood collected 24 hours after intraperitoneal (i.p.) injection of 100 µL PBS or anti-Gr-1 antibodies. Only the cells positive for Ly6C are depleted by anti-Gr-1 antibodies and higher expression of Ly6C results in increased depletion. (**I**,**J**): Percentage of Ly6G^{+} events in cardiac blood collected 72 hours after i.p. injection of 200 µL PBS, anti-Gr-1 antibodies or anti-ly6G antibodies demonstrates complete neutropenia. (**K**): The used DiD staining protocol resulted in a BMDM staining efficiency of almost 100%. Of note, due to spontaneous healing of the implanted tumor, one mouse from the Gr-1 control group (**B** and **E**) was omitted from analysis.

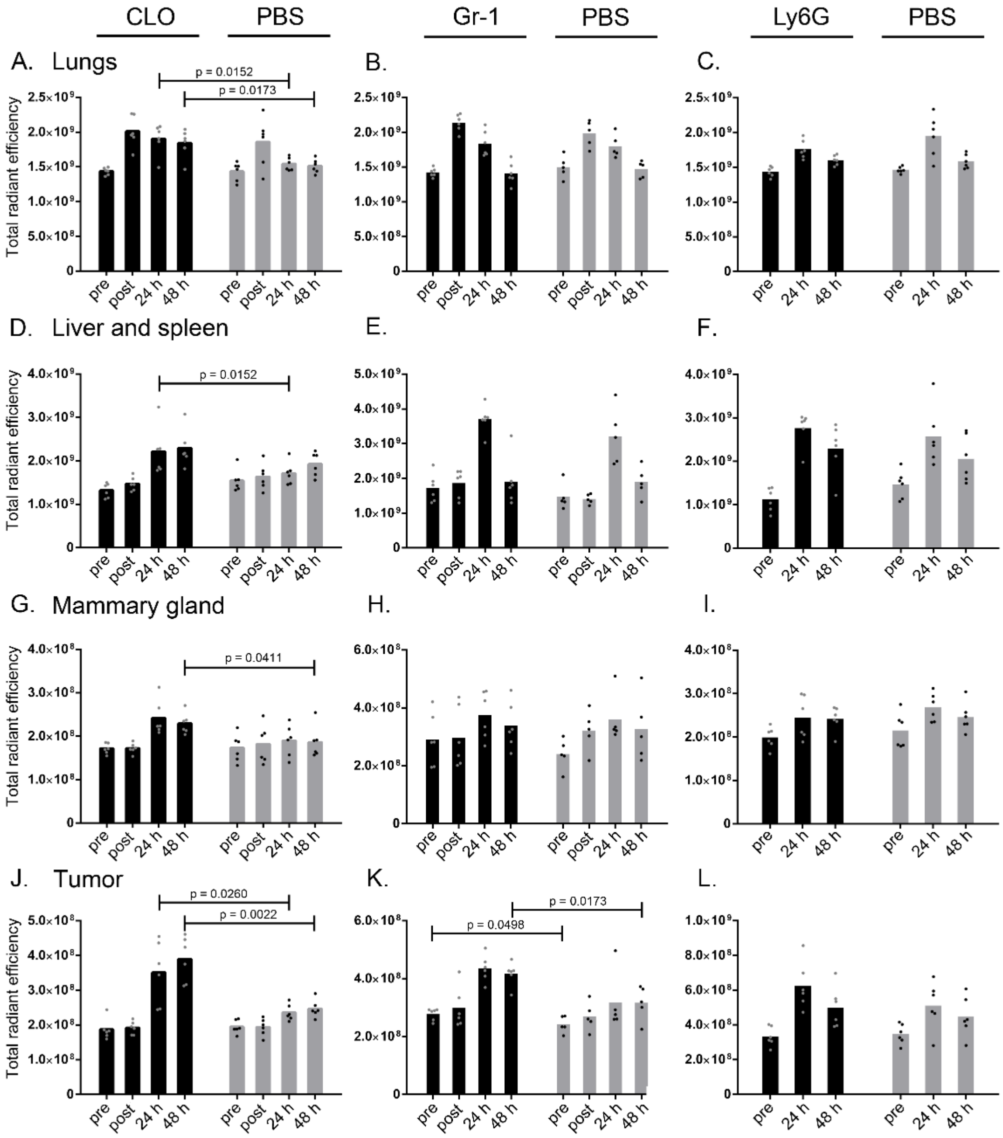

Figure S2. Total radiant efficiency (p/s/cm^{2}/sr) measured by in vivo macroscopic fluorescence imaging at different organs and the tumor immediately before (pre) and after (post) DiD-BMDM injection as well as at 24 hours and 48 hours after injection of DiD-labeled BMDMs. The performed experimental depletions (CLO, Gr-1 and Ly6G) are depicted in pair with their respective PBS controls. Directly after injection, exogenous BMDMs accumulated mostly in the lungs (**A**–**C**) and (except for mice pretreated with CLO-liposomes) disappear from this site over the next 48 hours. A redistribution of the DiD-BMDMs from the lungs to the liver-and-spleen region (**D**–**F**) is visible at 24 hours post injection. The mammary gland opposite to the tumor (**G**–**I**) is used as an indicator for increased background fluorescence due to circulating exogenous DiD-BMDMs. Only mice pretreated with CLO liposomes

exhibit significantly increased background fluorescence at 48 hours post BMDM injection. Tumors (**J**–**L**) of mice preatreated with CLO liposomes or anti-Gr-1 antibodies display significant higher amounts of DiD-BMDMs compared to PBS pretreated control mice. However, for the anti-Gr-1 experiment we have to remark that the mice in the Gr-1 group had a significant higher (p = 0.0498) initial background fluorescence ("pre-value") than their PBS controls. When taking this initial background fluorescence into account, anti-Gr-1 depletion does not increase the accumulation of BMDMs in the tumor (see Figure 1B). p-values below 0.05 are depicted. n = 6.

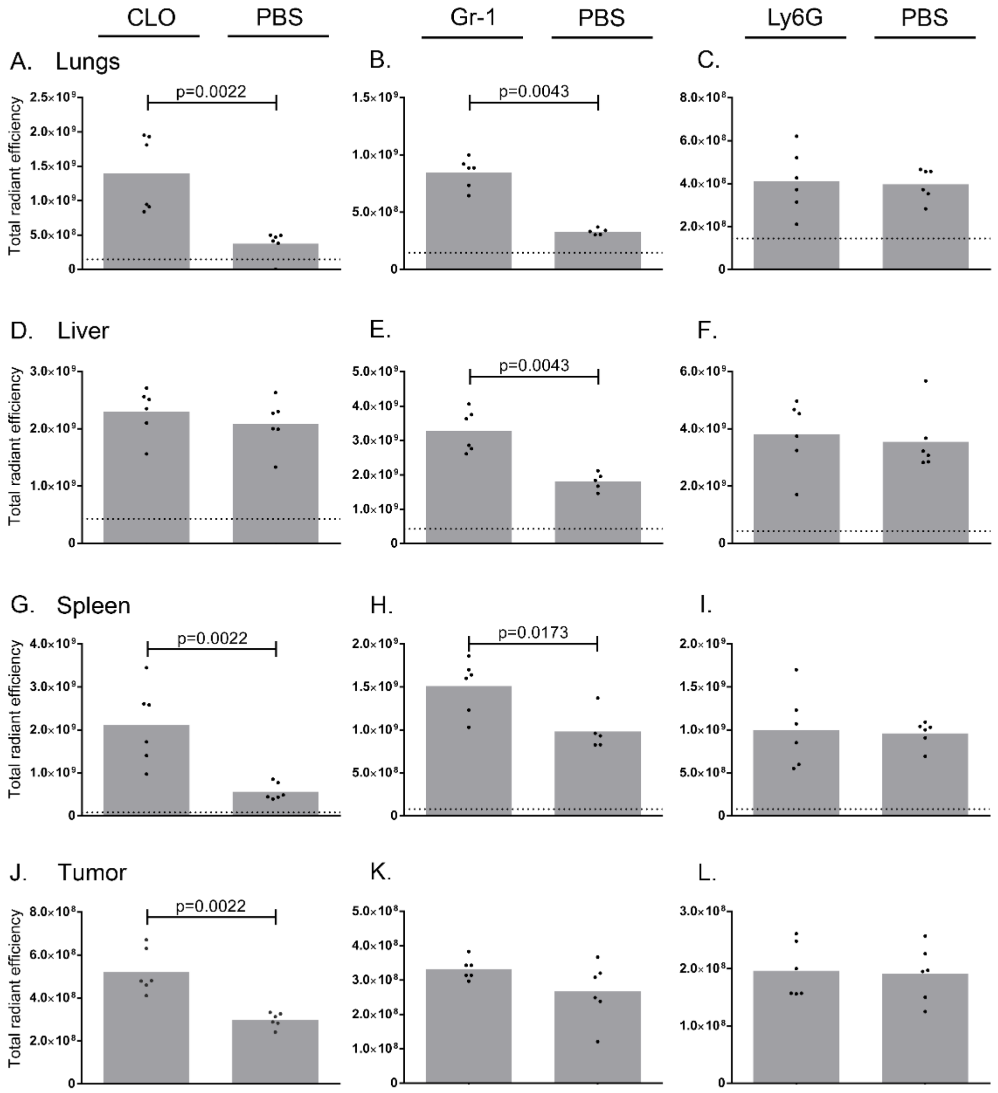

Figure S3. Total radiant efficiency (p/s/cm²/sr) of tumors and organs excised from mice euthanized 48 hours after injection of DiD-labeled BMDMs. The performed experimental depletions (CLO, Gr-1 and Ly6G) are depicted in pair with their respective PBS control. The tumor (**J**), lungs (**A**) and spleen (**G**) but not the liver (**D**) of mice pretreated with CLO liposomes demonstrate increased fluorescence. Conversely, all organs (**B**, **E** and **H**) except the tumor (**K**) of mice pretreated with anti-Gr-1 antibodies display increased fluorescence. Organs of mice pretreated with anti-Ly6G antibodies (**C**, **F**, **I** and **J**) demonstrate equal fluorescence as the control PBS-pretreated mice. p-values below 0.05 are shown. n = 6.

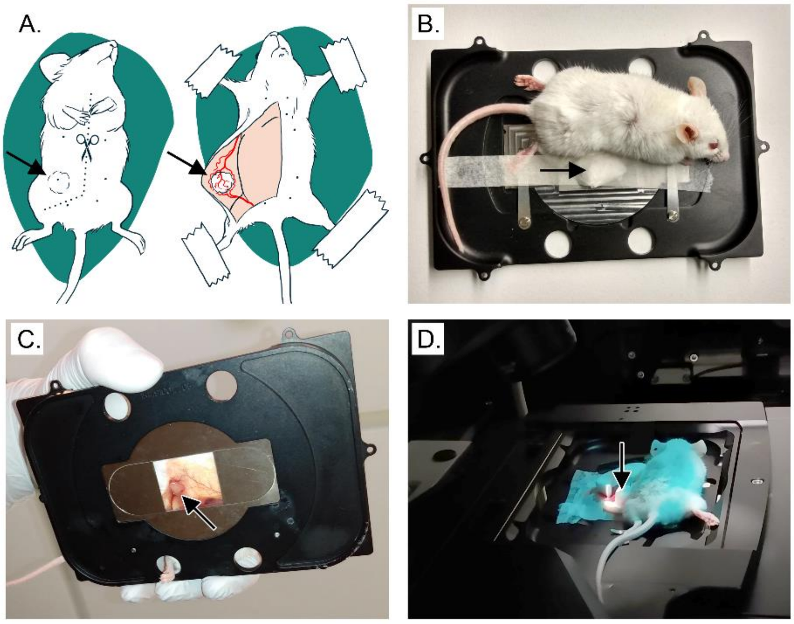

Figure S4. Setup of the intravital microscopy (IVM) procedure. (**A**) 1–1.5 × 10^6 4T1 tumor cells were inoculated in the right 4th mammary fat pad of female Balb/c mice. After 10–14 days, mice were anesthetized and the abdominal skin containing the tumor (indicated by the arrow) is carefully detached from the body while maintaining perfusion to prevent breathing movement during imaging. Next, the skin flap with the tumor is attached to a microscope platform that allows visualization of the tumor blood vessels and microenvironment from below (**B**). Image C shows the exposed tumor vasculature that is accessible for imaging on an inverted confocal fluorescence microscope. Lastly, the platform with the mouse is carefully positioned on the heated microscope stage after which confocal fluorescence imaging is started (**D**).

Video S1. Real time visualization of circulating DiD-labeled BMDMs in mammary fat pad tumors of PBS and CLO liposomes pretreated mice. Tumor bearing mice were first pretreated with either PBS or CLO liposomes. After 24 hours, 10^6 DiD-labeled BMDMs were injected and their appearance in the tumor microenvironment was monitored by intravital microscopy. Only a few circulating DiD-labeled BMDMs (red) can be seen 90 minutes after their injection in PBS-pretreated mice (PBS, left) whereas many BMDMs are still circulating, rolling and adhering even 6 hours after administration in the blood of CLO liposomes-pretreated mice (CLO, right). Hoechst (blue) and 40 kDa TMR-dextran (green) were systemically injected. Scale bar: 50 µm.

Video S2. Real time visualization of long-term circulating DiD-labeled BMDMs in mammary fat pad tumors of CLO liposomes-pretreated mice. Tumor bearing mice were first pretreated with CLO liposomes. After 24 hours, 10^6 DiD-labeled BMDMs were injected. Forty-eight hours after their administration, intravital microscopy of the tumor microenvironment revealed many DiD-labeled BMDMs still circulating. 2 MDa TMR-dextran (green) was systemically injected to visualize the blood vessels. Scale bar 50 µm.

Video S3. Z-stack (starting superficial) through a mammary fat pad tumor of a CLO liposomes-pretreated mouse that was injected with Hoechst (blue)/DiD (red) double labeled BMDMs. Tumor bearing mice were pretreated with CLO liposomes 24 hours before injection of the BMDMs. In addition, 10 hours before injecting BMDMs, 40 kDa (yellow) and 2 MDa (green) dextrans were systemically injected. About 30 minutes after double labeled BMDM injection, a Z-stack movie through the tumor was generated. The movie at the left side represents the merged image of the four individual channels on the right. Double labeled BMDMs can be seen in- and outside the blood vessels (angled V). However, sometimes only the Hoechst emission is visible (angled A) and some DiD-signal is associated with 40 kDa TMR-dextran-labeled endogenous macrophages (angled Y). Excitation with

a red laser can reveal superficial collagen (blue) via second harmonic effects (early frames). Note that the fluorescence signal disappears in a wavelength-dependent manner when scanning deeper in tissues (longer wavelengths penetrate deeper). Scale bar 50 μm.

Video S4. Z-stacks (starting superficial) through mammary fat pad tumors of PBS- and CLO liposomes-pretreated mice that were injected with DiD-labeled BMDMs. Pretreatment with PBS or CLO liposomes was performed 24 hours before BMDM administration. Endogenous macrophages and blood vessels were visualized by injecting 40 kDa TMR-dextran (shown in cyan) 4 hours before imaging. These Z-stack movies were generated about one hour after the injection of the labeled BMDMs. The movie clearly shows a much higher presence of DiD-labeled BMDMs (red) in the tumors of mice pretreated with CLO liposomes (CLO, right) compared to mice pretreated with PBS (left). These BMDMs are mainly seen in the extravascular compartment, but some intraluminal BMDMs can be seen as well. Scale bar: 50 μm.

© 2019 by the authors. Licensee MDPI, Basel, Switzerland. This article is an open access article distributed under the terms and conditions of the Creative Commons Attribution (CC BY) license (http://creativecommons.org/licenses/by/4.0/).
